# Characterization of the complete chloroplast genome of *Trailliaedoxa gracilis* (Rubiaceae)

**DOI:** 10.1080/23802359.2022.2155491

**Published:** 2023-01-01

**Authors:** Fengxiao Tan, Weixi Li, Jiaru Lü, Chengruo Pei, Qingwei Li, Youxin Jia, Jianwu Wang

**Affiliations:** a College of Natural Resources and Environment, South China Agricultural University, Guangzhou, China; b Guangdong Provincial Key Laboratory of Eco-Circular Agriculture, South China Agricultural University, Guangzhou, China; c School of Life Sciences, Sun Yat-sen University, Guangzhou, China; d College of Forestry and Landscape Architecture, South China Agricultural University, Guangzhou, China

**Keywords:** Chloroplast genome, endemic species, *Trailliaedoxa gracilis*, phylogenomic analysis, vulnerable species

## Abstract

*Trailliaedoxa gracilis* (Rubiaceae) is a Chinese endemic monotypic genus distributed in southwestern China. This study reported the complete chloroplast genome of *T. gracilis* assembled from Illumina sequencing reads. The chloroplast genome size is 152,407 bp, containing a single large copy (LSC) region of 82,957 bp, a short single copy (SSC) region of 17,936 bp, and a pair of inverted repeats (IRs) of 25,757 bp. A total of 127 genes were found, including 82 protein-coding genes, 37 tRNA genes, and eight rRNA genes. A phylogenetic analysis using the maximum likelihood algorithm revealed that *T. gracilis* belonged to the subfamily Ixoroideae and had the closest relationship with *Scyphiphora hydrophyllacea*.

*Trailliaedoxa* of the Family Rubiaceae is a monotypic genus endemic to China. *Trailliaedoxa gracilis* W.W.Sm. & Forrest 1917 grows in dry valleys at an altitude of 700–2300 m. The shrub species has a narrow distribution range and is endemic to southwestern China’s Jinsha and Red River Valley drainage areas (Wang et al. 2011). *Trailliaedoxa gracilis* is distinct from other species in Rubiaceae in its ericoid habit, pubescent styles, and schizocarpic fruits. The species is listed in China’s National List of Grade II Protected Plants (http://www.gov.cn/zhengce/zhengceku/2021-09/09/content_5636409.htm; accessed on 30 July 2022) due to its limited distribution and different taxonomic status (Wang et al. 2011). In addition, previous studies have also suggested that *T. gracilis* should be included in the list of critically endangered (CR) plants of China (Li et al. 2012; Jia et al. 2016). Insight into chloroplast genomics may help us understand a species’ evolutionary history, leading to better conservation efforts (Daniell et al. 2016). However, similar accounts are rare for this species, and the phylogenetic position of *T. gracilis* within the family Rubiaceae is uncertain (Robbrecht 1988; Kainulainen et al. 2013). To address this knowledge gap, we assembled the complete chloroplast genome of *T. gracilis* using high-throughput Illumina sequencing technology and conducted the phylogenomic analysis.

Total DNA was isolated from fresh leaves of an individual of *T. gracilis* collected from Kunming, Yunnan Province, China (102.7961°E, 26.3192°N). The specimen was deposited at the Herbarium of Sun Yat-sen University with the voucher number GX_18125 (contact: Yelin Huang, 894513420@qq.com). The Illumina Hiseq X Ten platform was used to carry out genome sequencing, with paired-end reads of 150 bp. We obtained a total of 10.67 Gb short sequences. The quality-trimmed clean reads were used for the chloroplast genome *de novo* assembly using GetOrganelle with default parameters (Jin et al. 2020). Finally, we used the GeSeq web server to annotate the genes (Tillich et al. 2017). The raw annotations were manually examined and adjusted to ensure accuracy.

Our study revealed that the complete chloroplast genome of *T. gracilis* was 152,407 bp in size, with 37.63% of the overall GC content. It contained a pair of inverted repeats (IRs) of 25,757 bp each, which were separated by a large single-copy region (LSC) of 82,957 bp and a small single-copy region (SSC) of 17,936 bp. The chloroplast genome contained 127 genes, including eight ribosomal RNA (rRNA) genes, 37 transfer RNA (tRNA) genes, and 82 protein-coding genes. Most of the genes were single copies in the SSC or LSC, whereas four rRNA genes, seven tRNA genes, and six protein-coding genes had two copies in the IRs.

To understand the phylogenetic position of *T. gracilis* in the family Rubiaceae, we downloaded complete chloroplast genomes of 31 species from the NCBI GenBank database. The species list included 30 species of the Rubiaceae family (including *T. gracilis*), and one species, viz. *Buddleja colvilei* (NC_042766.1), from the family Loganiaceae, was used as the outgroup. We found 86 genes, although not every gene was present in all 31 species. We used Geneious R11.0.4 to extract 58 protein sequence datasets (Kearse et al. 2012), which were shared by all 31 species. The sequences were aligned individually in MAFFT v7.037b using default parameters (Katoh and Standley 2013). The 58 individual alignments were then concatenated using SequenceMatrix-Windows-1.7.8 (Vaidya et al. 2011). A maximum-likelihood tree (bootstrap replications 1000), inferred by the best-fit model of JTT + F + I + G4, was constructed using IQ-TREE v2.0.3 (Minh et al. 2020). The phylogenetic tree showed that *T. gracilis* belonged to the subfamily Ixoroideae and had the closest relationship with *Scyphiphora hydrophyllacea* (Figure 1).

**Figure 1.**
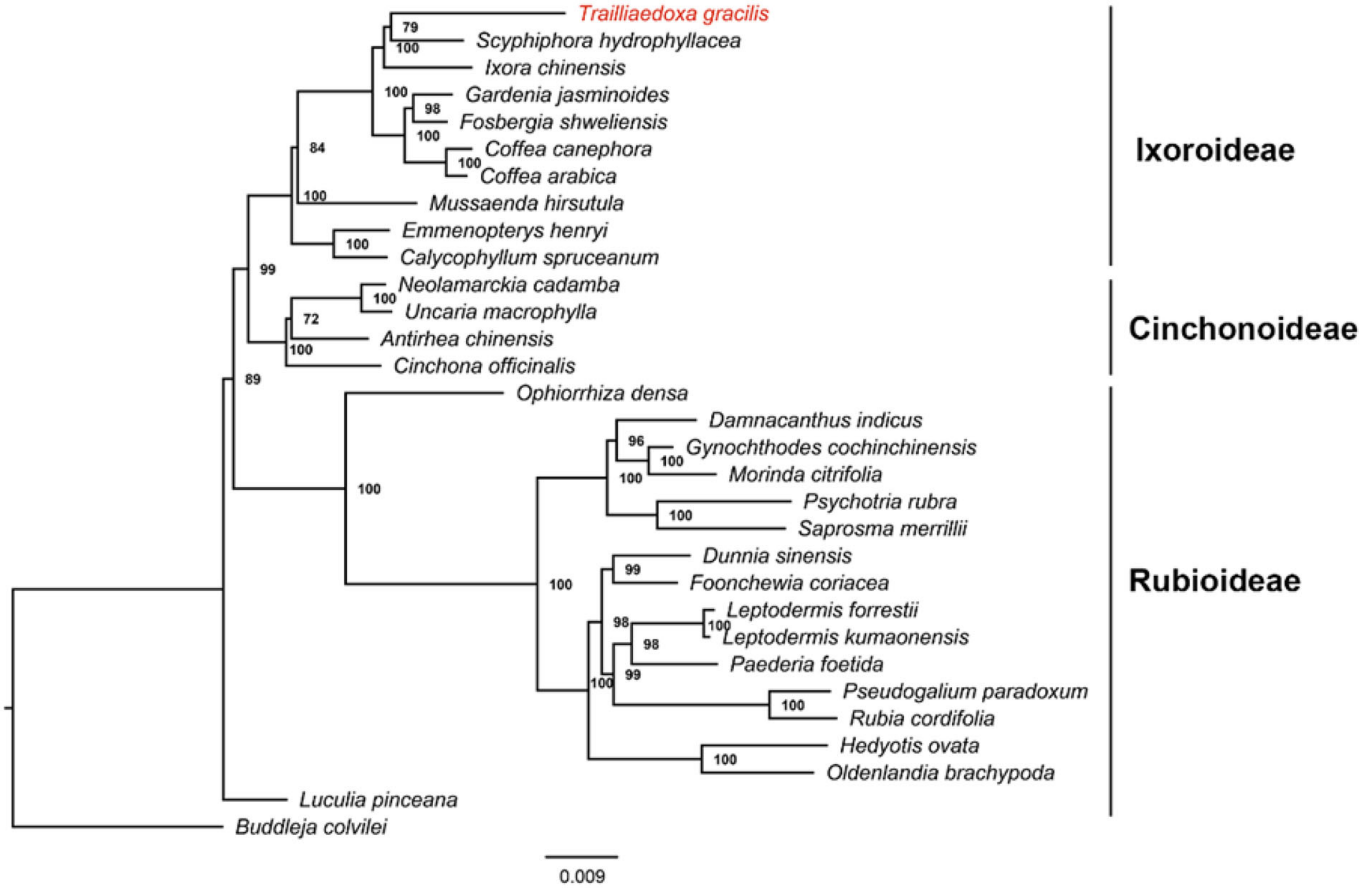
Maximum-likelihood tree based on the 58 concatenated protein sequences shared by all 31 species with complete chloroplast genomes (30 species from the family Rubiaceae to which *Trailliaedoxa gracilis* belongs and one species from the family Loganiaceae as an outgroup). Numbers in the nodes were bootstrap values from 1000 replicates. The position of *Trailliaedoxa gracilis* is shown in red. The GenBank accession numbers for each species and the citation sources for those sequences published are provided in supplemental Table S1.

It is the first study that characterized the complete chloroplast genome of *D. trichospermus*. The findings will provide a theoretical basis to understand the species’ evolution and phylogeny better. This information may also provide the genetic basis by which the conservation efforts can be strengthened, and the agronomic traits of this species can be enhanced.

## Supplementary Material

Supplemental Material

## Data Availability

The genome sequence data are available in GenBank under accession no. MK590999. The associated BioProject, SRA, and Bio-Sample numbers are PRJNA813343, SRR18249556, and SAMN26492387, respectively.
