# Supplementary material for: Characterization of the complete chloroplast genome of Trailliaedoxa gracilis (Rubiaceae)
Source: Mitochondrial DNA B Resour. 2023 Jan 1;8(1):1–3. doi: 10.1080/23802359.2022.2155491 (PMC9817128; doi:10.1080/23802359.2022.2155491)
Supplement: Supplemental Material [file TMDN_A_2155491_SM9625.docx]

| Table S1. The accession numbers for all 31 species used in the phylogeny reconstruction and the citation sources for those sequences published | | |
| --- | --- | --- |
| Species name | Accession number | Reference |
| *Antirhea chinensis* | MK102723.1 | - |
| *Calycophyllum spruceanum* | OK326865.1 | [1] |
| *Cinchona officinalis* | MZ151891.1 | [2] |
| *Coffea arabica* | MN370912.1 | - |
| *Coffea canephora* | KU500324.1 | - |
| *Damnacanthus indicus* | MW548283.1 | [3] |
| *Dunnia sinensis* | MN883829.1 | [4] |
| *Emmenopterys henryi* | KY273445.1 | - |
| *Foonchewia coriacea* | MT942688.1 | [5] |
| *Fosbergia shweliensis* | MT180075.1 | - |
| *Gardenia jasminoides* | MW160432.1 | [6] |
| *Gynochthodes cochinchinensis* | MW026443.1 | [7] |
| *Hedyotis ovata* | MK203877.1 | - |
| *Ixora chinensis* | MZ221832.1 | [8] |
| *Uncaria macrophylla* | MZ869757.1 | [9] |
| *Buddleja colvilei* | NC_042766.1 | [10] |
| *Leptodermis forrestii* | MT674522.1 | [11] |
| *Leptodermis kumaonensis* | MT674513.1 | [11] |
| *Luculia pinceana* | MW006821.1 | - |
| *Morinda citrifolia* | MN699649.1 | [12] |
| *Mussaenda hirsutula* | MK203878.1 | - |
| *Neolamarckia cadamba* | MG572117.1 | - |
| *Oldenlandia brachypoda* | MT767007.1 | - |
| *Ophiorrhiza densa* | MW683127.1 | - |
| *Paederia foetida* | OL449949.1 | [13] |
| *Pseudogalium paradoxum* | OK236360.1 | - |
| *Psychotria rubra* | MZ958829.1 | - |
| *Rubia cordifolia* | MN736957.1 | [14] |
| *Saprosma merrillii* | MK203879.1 | - |
| *Scyphiphora hydrophyllacea* | MN390972.1 | - |
| *Trailliaedoxa gracilis* | MK590999.1 | - |

1. Saldaña CL, Rodriguez-Grados P, Chávez-Galarza JC, Feijoo S, Guerrero-Abad JC, Vásquez HV, Maicelo JL, Jhoncon JH, Arbizu CI. 2022. Unlocking the Complete Chloroplast Genome of a Native Tree Species from the Amazon Basin, Capirona (*Calycophyllum Spruceanum*, Rubiaceae), and Its Comparative Analysis with Other Ixoroideae Species. Genes (Basel); 13(1):113.
2. Arbizu CI, Ferro-Mauricio RD, Chávez-Galarza JC, Guerrero-Abad JC, Vásquez HV, Maicelo JL. 2021. The complete chloroplast genome of the national tree of Peru, quina (*Cinchona officinalis* L., Rubiaceae). Mitochondrial DNA B Resour; 6(9):2781-2783.
3. Lou SM, Li H, Qin XM, Huang ZP, Jiang SY, Huang XY. 2021. The complete chloroplast genome of Damnacanthus indicus C.F.Gaertn. (Rubiaceae). Mitochondrial DNA B Resour; 6(3):1251-1252.
4. Zhang Y, Chen S, Xu X, Wang R. 2020. The complete chloroplast genome of *Dunnia sinensis* (Rubiaceae): a monotypic species endemic to Guangdong, China. Mitochondrial DNA B Resour; 5(1):814-816.
5. Zhang Y, Chen S, Xu X, Wang R. 2021. The complete chloroplast genome of *Foonchewia coriacea* (Rubioideae: Rubiaceae): a monotypic species endemic to Guangdong, China. Mitochondrial DNA B Resour; 6(1):156-157.
6. Zhang M, Chen WD, Li YY, Zhang C, Chai ZH, Li YF, Xie SW, Deng SY, Duan YF, Wang XR. 2021. Complete chloroplast genome of a wild-type *Gardenia jasminoides* ellis (rubiaceae) adapted to island climate. Mitochondrial DNA B Resour; 6(2):313-314.
7. Bautista MAC, Tao W, Zheng Y, Deng Y, Chen T, Miao S. 2021. Chloroplast genome organization and phylogeny of *Gynochthodes cochinchinensis* (DC.) Razafim. & B. Bremer (Rubiaceae). Mitochondrial DNA B Resour; 6(1):261-262.
8. Bian A, Lu L. 2021. The complete chloroplast genome of *Ixora chinensis* and phylogenetic relationships. Mitochondrial DNA B Resour; 6(11):3217-3221.
9. Zhang N, Song J, Liu H, Li F, Pan W. 2022. The complete chloroplast genome of *Uncaria macrophylla* Wall. (Rubiaceae) and its phylogenetic analysis. Mitochondrial DNA B Resour; 7(5):867-869.
10. Ge J, Cai L, Bi GQ, Chen G, Sun W. 2018. Characterization of the Complete Chloroplast Genomes of *Buddleja colvilei* and *B. sessilifolia*: Implications for the Taxonomy of Buddleja L. Molecules; 23(6):1248.
11. Zhang Y, Wang Z, Guo Y, Chen S, Xu X, Wang R. 2021. Complete chloroplast genomes of *Leptodermis scabrida* complex: Comparative genomic analyses and phylogenetic relationships. Gene; 791:145715.
12. Niu YF, Liu J. 2020. The complete chloroplast genome of *Morinda citrifolia* (noni). Mitochondrial DNA B Resour;5(1):377-378.
13. Wang W, Xu T, Song X, Chen C, Liu D, Han B, Yi S. 2022. The complete chloroplast genome of the medicinal plant *Paederia foetida* L. Mitochondrial DNA B Resour. 2022 Jul 7;7(7):1218-1220.
14. Zhao S, Liang H, Tang P, Muchuku JK. 2022. A complete chloroplast genome of Rubia yunnanensis Diels (Rubiaceae), a traditional Chinese herb endemic to China. Mitochondrial DNA B Resour; 7(8):1466-1467.
